# Nascent Folding of Proteins Across the Three Domains of Life

**DOI:** 10.3389/fmolb.2021.692230

**Published:** 2021-06-07

**Authors:** Mateusz Chwastyk, Marek Cieplak

**Affiliations:** Institute of Physics, Polish Academy of Sciences, Warsaw, Poland

**Keywords:** ribosome exit tunnel, geometry, molecular dynamics, coarse-grained models, protein folding, proteins with knots

## Abstract

We study the nascent behavior of three model coarse-grained proteins in six rigid all-atom structures representing ribosomes that come from three domains of life. The synthesis of the proteins is implemented as a growth process. The geometry of the exit tunnel is quantified and shown to differ between the domains of life: both in volume and the size of constriction sites. This results in different characteristic times of capture within the tunnel and various probabilities of the escape. One of the proteins studied is the bacterial YibK which is knotted in its native state. A fraction of the trajectories results in knotting and the probability of doing so is largest for the bacterial ribosomes. Relaxing the condition of the rigidness of the ribosomes should result in a better avoidance of trapping and better proper folding.

## Introduction

**FIGURE 1:**
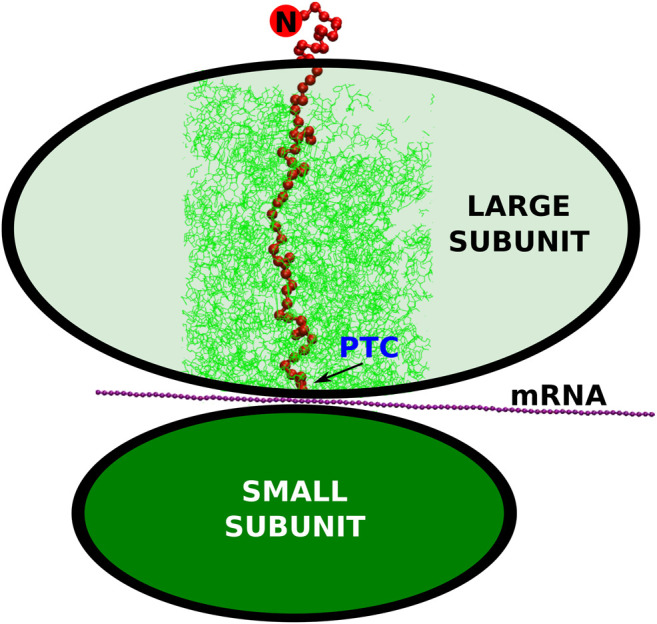
The schematic representation of the small and large ribosomal subunits (the green ovals). The arrow points to the peptidyl-transferase center where the conversion of the nucleic acid genetic information (mRNA) into the polypeptide (the red string) takes place. The cylindrical cut-out, considered in our simulations, is marked by the thin, green lines. The N-terminus of the synthesized protein is marked by the red circle with the letter “N”.

Ribosome is a biomolecular nanomachine that performs protein synthesis at its peptidyl-transferase center (PTC) as directed by an mRNA template. The schematic picture of the protein being created by the ribosome is presented in Figure 1. In terms of the evolution, the PTC has been recognized as the earliest part of the ribosome (Prosdocimi et al., 2020). Ribosome itself is an aggregate made of 2–6 RNA chains and around 50 proteins comprising altogether between 100,000 and 220,000 atoms. Six examples of the ribosomal structures, together with some parameters of their description, are listed in Table 1. Ribosomes are involved in the regulation of translation and they influence the folding process (Kaiser et al., 2011). The synthesis takes place at the rate that depends on the domain of life. For prokaryotes, it is about 20 amino acids (Young and Bremer, 1976) and for eukaryotes four amino acids (Boström et al., 1986; Ingolia et al., 2011). The PTC secretes the nascent protein into the exit tunnel that ends with a “mouth” that opens into the surrounding solvent. After the detachment from the center, the protein escapes the ribosome in less than 1 ms (Bui and Hoang, 2020; Nissley et al., 2020). The movement of the protein toward the solvent gets started at the PTC and then it is influenced mostly by diffusion, interactions with the walls of the tunnel, and the gain in the entropy associated with the escape. The walls of the tunnel are rough and its diameter varies between 10 and 20 Å (Voss et al., 2006; Cabrita et al., 2010; Frank and Gonzalez, 2010). It has been established (Melnikov et al., 2012; Dao Duc et al., 2019; Liutkute et al., 2020) that the very geometry of the tunnel depends on the domain of life. In particular, we find that the diameter in the bacterial ribosomes can reach even 30 Å. This happens at the branching points in the tunnel.

**TABLE 1:** Six ribosomal structures considered in our analysis.

	PDB	Organelle	MM [kDa]	*P*_*ch*_/*RNA*_*ch*_	*V*_*T*_ (*R* = 4*Å*) [Å^3^]	*V*_*T*_ (*R* = 3*Å*) [Å^3^]	*S*_*T*_/*V*_*T*_ [1/Å]	*C*_*at*_/*R*_*at*_
bacteria	5NJT	*B. subtilis* Beckert et al. (2017)	2,111.70	48/3	27145 ± 407	34920 ± 490	0.33	11971/133097
	5AFI	*E. coli* Fischer et al. (2015)	2,302.71	52/6	25628 ± 255	36295 ± 779	0.33	12330/152717
archaea	4V9F	*H. marismortui* Gabdulkhakov et al. (2013)	1,486.40	31/2	11333 ± 132	15188 ± 156	0.41	14499/103831
	4V6U	P. furiosus Armache et al. (2012)	2,602.99	63/5	16446 ± 216	24348 ± 362	0.38	13052/173979
eukarya	6EK0	H. sapiens Natchiar et al. (2017)	3,842.38	76/5	16822 ± 246	26838 ± 248	0.41	12702/219596
	5XY3	T. vaginalis Li et al. (2017)	1713.54	40/3	20043 ± 175	25213 ± 229	0.40	11680/107145

The bacterial ribosomes considered correspond to structures PDB:5NJT and PDB:5AFI that originate from *B. subtilis* and *E.coli* respectively. The archaeal ribosomes were from *H. marismortui* and from *P. furiosus* (PDB:4V9F and PDB:4V6U) The last two eukarya ribosomes are eukaryotic and they come from *H. sapiens* (PDB:6EK0) and from *T. vaginalis* (PDB:5XY3). The second column shows the PDB code of the particular structure. The third column lists the source organism (italicized) and the references for the presented data. The fourth column lists the total structure molecular weight; the fifth column lists the number of unique proteins and, after the slash of the, nucleic acid chains. The sixth column lists the volume of the exit tunnel calculated with the probe radius *R* = 4Å and the seventh for *R* = 3Å. The eighth column lists the surface-area-to-volume ratio for the exit tunnels. The last column lists the fraction of the atoms within the cylindrical cut-out, considered in our simulations, to the total number of atoms that make the full structure of the ribosome. The two numbers differ by an order of magnitude.

Here, we present results of a theoretical study in which we probe the impact of the nature of the ribosome on protein folding in a coarse-grained model. Specifically, we consider six ribosomes from three domains of life, bacteria, archea and eukarya, and elucidate the difference in behavior of three model bacterial proteins in the six ribosomal tunnels. The bacterial ribosomes considered correspond to structures PDB:5NJT (Beckert et al., 2017) and PDB:5AFI (Fischer et al., 2015) that originate from *B. subtilis* and *E. coli* respectively. The remaining structures and their origins are listed in Table 1. The first protein selected is the streptococcal protein G (Gronenborn et al., 1991) (PDB:1GB1) of 56 residues that is a common object of simulational studies. The second is YibK (Lim et al., 2003) (PDB:1J85) derived from *Haemophilus influenze*. YibK consists of 156 residues. The reason to consider it is that its native structure contains the deep trefoil knot between sites 75 and 119. It means that the backbone of the protein entangles themselves in to a knot. In the case of deeply knotted proteins, the transient state during the knotting process is often a slipknot. It is a conformation in which one of the protein termini adopts a hairpin-like conformation that threads a loop formed by the remainder of the chain (Faísca, 2015). The ribosomal action has been proposed (Chwastyk and Cieplak, 2015) to solve the puzzle of how the deeply knotted proteins form. An additional mechanism that may enhance knotting still further involves confinement generated by post-translational action of chaperonins (Takagi et al., 2003; Mallam and Jackson, 2011; Soler et al., 2016; Zhao et al., 2017; Especial et al., 2019; Chwastyk et al., 2021). It should be noted that the ribosome itself also provides a confining space that may favor formation of secondary structures (Elcock, 2006). The last protein we consider is Trp-Cage miniprotein (PDB:1L2Y) (Neidigh et al., 2002). It is composed of 20 amino acids and its small size allows it to fold near the PTC.

Theoretical studies of cotranslational folding, especially when knotting is involved, should start by considering simple coarse-grained structure-based models (see, e.g., ref (Sikora et al., 2009)) as they introduce a bias toward the native state. In our previous paper (Chwastyk and Cieplak, 2015), the mouth of the ribosome has been represented as an infinite repulsive plate which grows proteins by starting from the N-terminus. The plate has turned out to be positioning the planar knot loop of YibK (between sites 75–95) in a way that allows for formation of a C-terminal slipknot and then threading of the C-terminus through the loop on detachment. The effectiveness of this mechanism depends on the scheme to derive the contact map and it increases with time separation between the successive events of the emergence of new residues, *t*_*w*_. The longest used was 5,000 τ where τ is of order 1 ns—the characteristic time scale of the CG simulation (Sikora et al., 2009). A better approach is to implement a steady growth at the PTC (Hoang and Cieplak, 2000; Krobath et al., 2013; Bui and Hoang, 2020).

Another simple model (Dabrowski-Tumanski et al., 2018) represents the exit tunnel as a smooth funnel-like potential. It is combined with an axial force that acts on the fully formed sequence placed near the PTC and pushes it toward the mouth. Necessarily, this force must induce an acceleration which is likely spurious. This approach was used for a large deeply knotted protein Tp0624 of 421 residues. The crucial ingredient in achieving high effectiveness in knotted folding was adding attractive centers at the mouth of the exit tunnel. However, we find that such centers generate trapping at the mouth.

Making comparisons between the domains of life, however, cannot be based on the ribosome models that are just generic. Here, we consider a coarse-grained model (Chwastyk et al., 2021) in which the growth takes place at the PTC and the tunnel has the shape that is determined by the structure file of the ribosome so that it is sensitive to the species (In ref. (Chwastyk et al., 2021), we have discussed the bacterial structure PDB:4V4J from *Thermus thermophilus*.). We first compare the geometries of the tunnels arising in the six ribosomes of Table 1 and show how they differ across the domains of life. We then compare the folding processes and show that the bacterial ribosomes enhance the folding and knotting processes stronger than the other ribosomes. The relatively small average diameters of the archaea and eukarya ribosomal exit tunnels make the protein growing process difficult. The most efficient folding process happens within the ribosomal structure which is natural for a given protein.

### The Geometry of the Ribosomal Exit Tunnels

**FIGURE 2:**
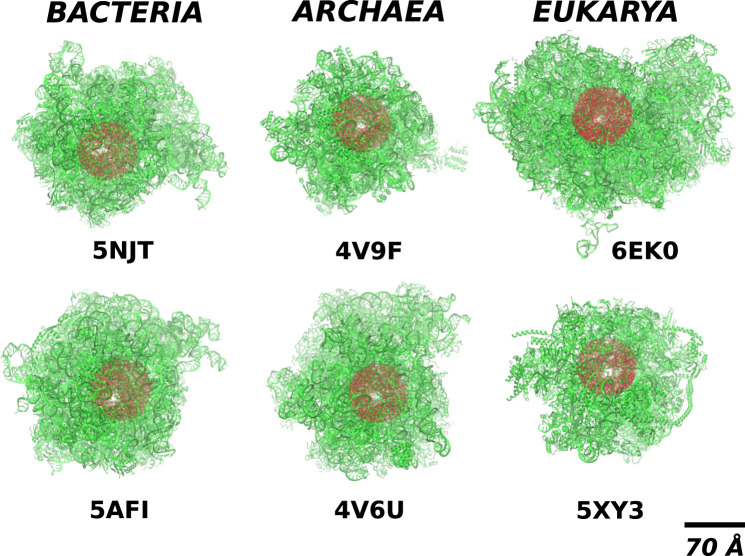
The location of the cylindrical cut-out (the red shadow) of radius *R*_*R*_ = 70Å within the full structure of ribosomes (the green colour) from different domains of life. The white spot within the red blob shows the position of the exit tunnel.

**FIGURE 3:**
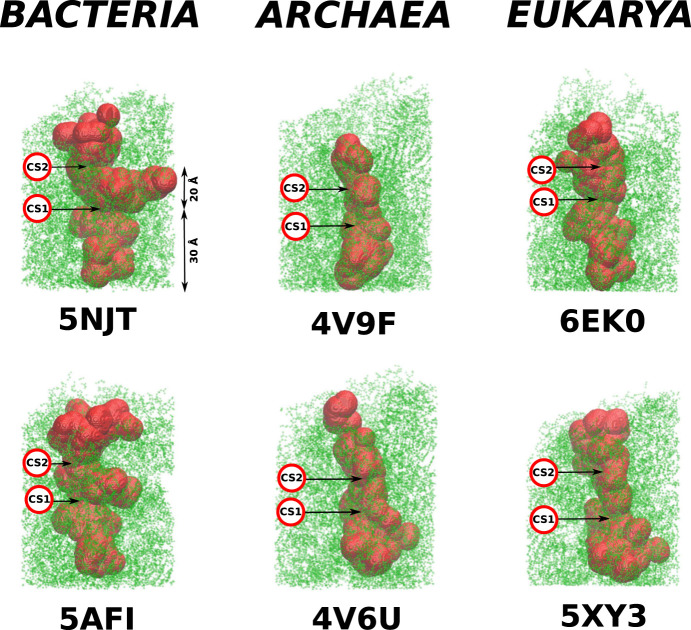
Structures of the cylindrical cut-outs around the ribosomal exit tunnels from the three different domains of life. Each of the six panels shows the all-atom representation of the cylinder. The colors are meant just to generate a sense of perspective. The structures shown below the main picture represents the very exit of the cylinder. Up to five colors are used to indicate the vertical positions. The ball-like structure on the right of the cylinder represents the top view of the exit mouth.

**FIGURE 4:**
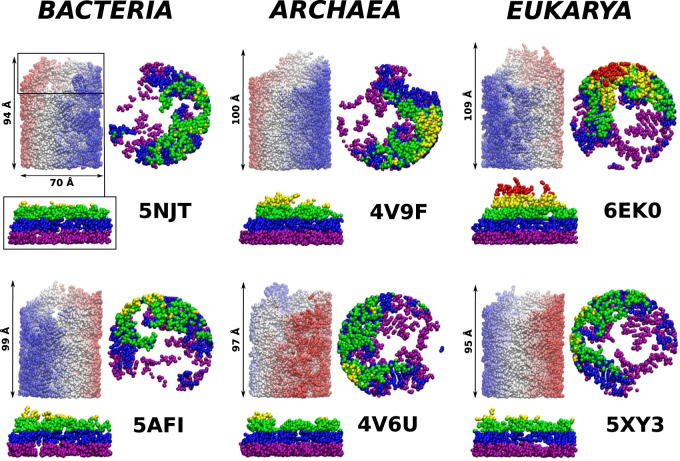
The ribosomal exit tunnels (the red shape) detected by the SPACEBALL algorithm (Chwastyk et al., 2014; Chwastyk et al., 2016a; Chwastyk et al., 2016b) within the ribosomal structures from different domains of life. The arrows point to the constriction sites (CS1 and CS2). The green shadow represents the all atoms that are present in the cut-out part of the ribosomes. The PTC is always at the bottom of each panel.

The complexity of the full ribosomal structure makes it hard to include all of the atoms in the simulations. Thus we limit the number of relevant atoms to a cylindrical cut-out of radius *R*_*R*_ = 70 Å around the ribosomal exit tunnel as illustrated in Figures 2, 3. The cylinder originates at the plane that goes through the PTC. It is oriented towards the mouth of the ribosome as shown in Figure 2 against the background of the full ribosome. The structures around the ribosomal exit tunnel incorporated in our simulations are presented in Figure 4. Each cylinder is composed of rRNA and ribosomal proteins atoms. The specific number of nucleic acid and protein chains are listed in Table 1. Here, we consider these atoms to be fixed rigidly during the dynamics but an improved model should allow for their flexibility.

The location of the PTC was determined based on the data presented by Dao Duc et al. (2019)—it is found at the extension of the L22 and L4 proteins (Trylska, 2009; Dao Duc et al., 2019). In order to identify the tunnels and their volumes, we used the SPACEBALL algorithm (Chwastyk et al., 2014; Chwastyk et al., 2016a; Chwastyk et al., 2016b). The algorithm involves sending probing particles along main directions of a grid (at ten rotations of the ribosome) and checking which grid sites are accessible. Usually, one takes the radius of the probing particles to be 1.4 Å which is equal to the diameter of the water molecule. Here, we use primarily the radius of 4 Å in order not to penetrate the all-atom representation of the walls of the tunnel, but we also make comparisons to calculations done with the radius of 3 Å.

Our calculated volumes are somewhat smaller in comparison to the results obtained by Dao Duc et al. (2019). They got (3.85 ± 0.37)×10^4^ Å^3^ by considering 10 bacteria and (2.78 ± 0.13)×10^4^ Å^3^ for nine eukarya. When we decreased the radius of our probe to 3 Å we got very similar results, listed in Table 1. The difference in our algorithm is that we do not specify the position of the tunnel before undertaking the volume calculation. In addition, we make rotations of the grid. In the first step, Dao Duc et al. (2019) search for the tunnel position by using MOLE 2.0 (Sehnal et al., 2013) program and then implement HOLLOW (Ho and Gruswitz, 2008) approach to determine the volume. The HOLLOW approach is very similar to our method but for well defined structure. This allows for the usage of smaller probes and thus to obtain larger volumes.

We observe that the bacterial ribosomes are associated with tunnels of the largest volume and are thus expected to obstruct the motion toward the exit the least. The SPACEBALL-derived tunnel spaces are shown in Figure 4 in the red color. Despite the existence of pronounced side channels, the bacterial tunnels come with much fewer regions that are difficult to access. This was confirmed just by looking at the detected exit tunnel and by calculating the surface-area-to-volume ratio for the exit tunnels (see Table 1). The smallest value of this parameter was obtained for the bacterial exit tunnels and the largest one, for the eukaryotic tunnels.

The details of the cylindrical structures are shown in Figure 3. They are focused on the mouth regions and demonstrate that the openings are fairly irregular—they are not smooth funnel-like surfaces. The panels in Figure 3 also show the vertical distances from the PTC plane to the most distant atoms. These distances vary between 94 Å and 109 Å and do not distinguish between the domains of life.

As in Dao Duc et al. (2019), we find that there are several constriction sites (CS) in the ribosomes. The first of these, CS1, is located around 30 Å from the PTC for each of the six ribosomes studied. The second, CS2, is at 20 Å farther away from CS1. The width of CS2 depends on the domain of life: it is narrow in the eukaryotic case (the radius is around 8 Å), a bit wider for the archaea ribosomes (the average radius is around 11 Å), and still wider for the bacterial case (the average radius is around 15 Å). The locations of the CS1 and CS2 are marked in Figure 4.

## Cotranslational Folding

### Description of the Molecular Dynamics Model

The protein is modeled within the structure-based approach, as described in refs (Sulkowska and Cieplak, 2008; Sikora et al., 2009). with a chirality potential being responsible for the backbone stiffness. The contact interactions are selected by using the overlap criterion (Wołek et al., 2015) between the atoms of the residues as determined in the fully folded native state. The contacts correspond to the potential wells between the effective residues are located at the α-C atoms. The depth of the wells is denoted as ε. The remaining interactions are softly repulsive with the characteristic length of 4 Å. When one starts from an extended conformation and studies folding then the process is declared accomplished if all contacts get established. i.e., the distance between the residues involved becomes smaller than 1.5 σ, where σ denotes the width of the corresponding well. The temperature is controlled by random forces and the room temperature, *T*_*R*_ is around 0.35*ε*/*k*_*B*_. Each of the ribosomal atoms is a source of the soft repulsive potential that is cut at 4 Å and has the amplitude of ε.

Dynamically, the bottom of the cylinder is represented by a repulsive wall with the potential $\frac{3\sqrt{3}}{2}\varepsilon {\left(\sigma_{0}/z\right)}^{9}$, where *z* denotes the distance away from the plate and $\sigma_{0}=4\cdot 2^{-1/6}$. This wall prevents making any backward steps. The models that incorporate the sequential growth consider it taking place either at the PTC or, effectively, at the mouth.

The most common practice is to place a fully synthesized chain near the PTC (Frank and Gonzalez, 2010; Elcock, 2006; Dabrowski-Tumanski et al., 2018; Nissley et al., 2020) and then to monitor folding (Nilsson et al., 2015; Kudva et al., 2018; Bock et al., 2018). The exit through the mouth can be helped computationally by switching from a CG simulation to a steered all-atom molecular dynamics approach with a steady motion of a pulling cantilever (Nissley et al., 2020). Another method is to apply a constant force (Dabrowski-Tumanski et al., 2018). Here, we incorporate the sequential growth at the PTC. Each of the amino acids emerges with some time interval after the previous one created earlier. The direction of the amino acid motion is given by the repulsive potential accelerating the created bead toward the exit of the tunnel. Since the mRNA is translated from the 5’ to 3’ ends, the proteins are synthesized from the N terminus to the C terminus, so the N terminus emerges first, as presented in Figure 1. This method is similar to the one considered in ref (Bui and Hoang, 2020). There are several differences, however: 1) all protein and RNA atoms of the walls provide repulsion (not just the α-C atoms), 2) the backward motion is prevented by the repulsion from the bottom wall, 3) the growth is implemented in a quasi-continuous fashion, 4) in one variant of the model, used for the YibK protein, we introduce electrostatics-mimicking contacts at the mouth of the ribosome, similarly to Dabrowski-Tumanski et al. (2018). The strength of these contacts is the same as in the case of the intramolecular contacts. We declare that the contacts can be created between two amino acids with opposed electric charge. We have found that the presence of such contacts do not impact the knotting process, but impedes the dissociation of the protein from the ribosome, so the results presented here have been obtained without these attractive contacts.

**FIGURE 5:**
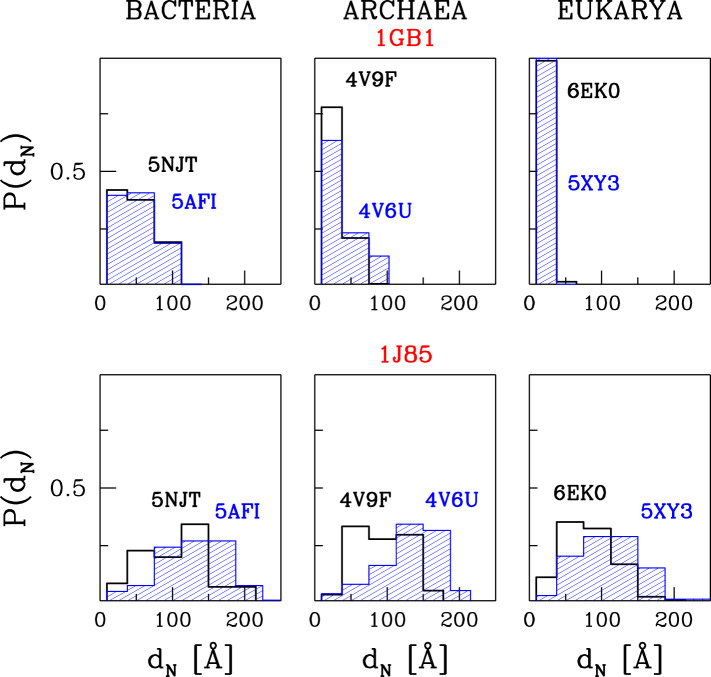
The histograms of the distances between PTC and the N-terminus after time *t*_*E*_ of simulations at *T*_*R*_ = 0.35*ε*/*k*_*B*_ and with *t*_*w*_ = 100*τ*. The results are for three different domains of life. The upper panels are for 1GB1, with *t*_*E*_ = 50000*τ*, and the lower ones for 1J85, with *t*_*E*_ = 150000*τ*.

We represent our results as in Figure 5. It shows the histograms of distances of the N-terminal points of the proteins awya from the PTC. The bin size was chosen so that the first bin corresponds to the trajectories that are stuck between the PTC and CS1, the second one—between CS1 and CS2 and the others—the trajectories that resulted in proteins leaving the ribosome.

### Protein G

**FIGURE 6:**
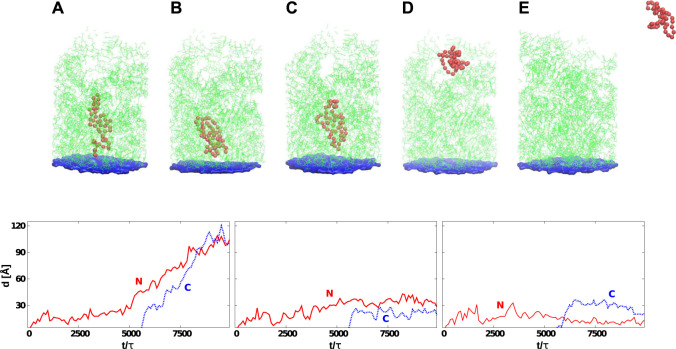
**Top:** Six snapshots of protein G (the red color) at various stages of its development that start at its synthesis **(A)** and, in many cases, end with the detachment **(E)**. The average time needed by the ribosome to release the protein depends on *t*_*w*_. The exit tunnel (in green) corresponds to the eukaryotic structure PDB:6EK0. The blue plane represents the origin of the repulsive potential. The PTC is located at the center of the plane. In **(B)**, the protein is stuck permanently at the CS1. In **(C)**, the jamming is at CS2. In **(D)**, the protein has reached the mouth of the tunnel. **Bottom:** Distances between the PTC and the N-terminus (solid-red line) or C-terminus (dotted-blue line) as a function of time. The left panel corresponds to the situation presented in **(D)** (protein is leaving the ribosome). The graph in the center corresponds to the situation shown in **(C)** (the protein gets stuck at CS2). The last graph shows the situation corresponding to **(B)** (the protein is stuck between PTC and CS1). In all cases, the C-terminus gets released after the synthesis of the 56-residue protein which, for *t*_*w*_ = 100τ, corresponds to 5,600 τ.

The upper panels of Figure 5 are for protein G. The temperature is equal to *T*_*R*_ and *t*_*w*_ is set to 100 τ, similarly to ref. (O'Brien et al., 2011). The panels show that the most difficult part of the movement of the growing protein is to pass through constriction CS1. For the two eukaryotic ribosomes, almost all of the trajectories get stuck at CS1. In the case of the two archaea ribosomes, this happens with 60–80% of the trajectories and around 20% get stuck at CS2. However, the bacterial ribosomes offer an easier passage for protein G: 40% of the trajectories stop at CS1, another 40% at CS2. Thus around 20% reach the mouth of the ribosome. Folding takes place after the exit and the median folding time of protein G in the 5NJT and 5AFI ribosomes is 7747*τ* and 7566*τ* respectively. An illustration of the various stages of the process is given in Figure 6. The folding process is considered to be accomplished when all native contacts are established for the first time (the distance between the corresponding α-C atoms is smaller than 1.5 σ where σ is the length parameter associated with the potential well. We considered 90 folding trajectories for each of the ribosomes.

When *t*_*w*_ is increased to 500*τ*, the number of trajectories that get stuck at the CS1 becomes similar for the two eukaryotic ribosomes and for the 4V6U archaea ribosome. However, it gets halved for the bacterial ribosomes while it increases from 80 to 100% for 4V9F (archaea). A further increase in *t*_*w*_ results in an increased probability of stopping at CS1 because there is more time to penetrate the exit tunnel nooks. The eukarya ribosomes do not allow for proteins leaving the ribosomal exit tunnel. We expect that when the walls are made flexible the protein will have a better chance of negotiating the constrictions at any *t*_*w*_, but this remains to be demonstrated. These results are based on 60 trajectories for each ribosome.

### Protein YibK

We now consider the knotted YibK protein. The knot ends are at LEU-75 and LYS-119 in the native state. We examine the impact of the full ribosomal structure on the knotting process. Unfortunately, it was impossible to reach *t*_*w*_ = 5000*τ*, as in ref (Chwastyk and Cieplak, 2015). that this time gives the highest value of the percentage-wise success of reaching the properly knotted folded conformation. *t*_*w*_ longer than 100*τ* decreases the number of trajectories with the full exit of the protein. To be able to examine the knotting process we did a numerical trick and we allowed for a fast growth (with *t*_*w*_ = 100*τ*) at the beginning of the protein synthesis and then switched to a slower process with *t*_*w*_ = 1000*τ* when the process reached LEU-75. This trick generates between 63 to 70 trajectories out of 100, depending on the ribosome, that manage to escape from the tunnel.

For this set of successful trajectories, about 10% resulted in formation of non-native temporal knots with one knot end at LYS-119. Another ∼10% resulted in temporal knots with improperly located knot ends. We expect, however, that an extension of the simulation will eventually move the ends to the proper locations. The most interesting situation is observed for the 5AFI ribosome. Only in this case, 1% of 1,000 trajectories resulted with the properly folded and knotted conformations. The knotting mechanism was based on a slipknot transient conformation, the same as observed in ref. (Chwastyk and Cieplak, 2015). In addition, 10% of the trajectories resulted in one temporal knot end located at LYS-119 and 5% of the trajectories with temporal knot elsewhere. Again, an extension of the simulations is expected to move the knot ends to their native locations. When we increased the temperature from *T*_*R*_ to 0.45 *ε*/*k*_*B*_ the total number of trajectories that escape the tunnel got decreased, but the success in folding and knotting was similar to the one at *T*_*R*_.

### Trp-Cage Miniprotein

In the case of the very short protein (1L2Y) which we also examined, all of the 100 trajectories got stuck at the CS1. Nevertheless, there is enough space between PTC and CS1 for this short protein to accomplish correct folding. This happens for all trajectories and the average folding time is 1979*τ*. The average here is over the trajectories and the six ribosomes.

## Conclusion

We have demonstrated the existence of differences in the dynamical behavior of nascent proteins across the domains of life. These differences result from the dissimilarities in the ribosomal geometries. The tight eukarya ribosomal exit tunnels impede the protein movement towards the exit. The wider constrictions sites found in the two other domains of life allow for proteins for an easier squeezing through away from the PTC. It is important to mention that the percentage-wise success of reaching the properly knotted and folded conformations was found to be the highest for 5AFI ribosome that originates from the *E. coli* bacterium. The YibK protein is also bacterial (*Haemophilus influenzae*). The two bacteria have one common ancestor (de Rosa and Labedan, 1998) which suggests that the 5AFI ribosome is evolution-related to the one that makes YibK. It is thus natural to consider YibK in this ribosome and expect the best folding results in this case.

If the longer sequences manage to exit the mouth and start making globular structures then this process, in principle, should be helped by attractive contacts at the mouth by generating an extracting mechanism. However, we find such contacts to be disruptive to the process because they also capture the extracted protein at the mouth. The reason why the attractive patches at the mouth appear to promote knotting in Dabrowski-Tumanski et al. (2018). may be related to the fact that the bulk driving force used also eliminates the capture. This point needs to be elucidated further.

In our current model, the ribosomal molecules are considered to be rigid. Relaxing this condition should result in better escape rates and possibly also in a stronger knotting success. Moreover, it will allow for usage of the all-atom protein model, because right now the side chains block the protein that attempt to squeeze through away from the PTC. The flexibility can be introduced either by connecting the atoms to fixed centers by harmonic springs or by coarse-graining the RNA and protein chains of the ribosome (Kudva et al., 2018). We hope to address these issues in future research. We also plan to use the Dynamical Structure-Based model (Mioduszewski and Cieplak, 2018) to consider the exit times for selected intrinsically disordered proteins and study their behavior in the tunnel.

## Data Availability

The raw data supporting the conclusions of this article will be made available by the authors, without undue reservation.

## References

[B1] Armache J.-P. Anger A. M. Márquez V. Franckenberg S. Fröhlich T. Villa E. (2012). Promiscuous Behaviour of Archaeal Ribosomal Proteins: Implications for Eukaryotic Ribosome Evolution. Nucleic Acids Res. 41, 1284–1293. 10.1093/nar/gks1259 23222135 PMC3553981

[B2] Beckert B. Abdelshahid M. Schäfer H. Steinchen W. Arenz S. Berninghausen O. (2017). Structure of the Bacillus Subtilis Hibernating 100S Ribosome Reveals the Basis for 70S Dimerization. EMBO J. 36, 2061–2072. 10.15252/embj.201696189 28468753 PMC5509997

[B3] Bock L. V. Kolář M. H. Grubmüller H. (2018). Molecular Simulations of the Ribosome and Associated Translation Factors. Curr. Opin. Struct. Biol. 49, 27–35. 10.1016/j.sbi.2017.11.003 29202442

[B4] Boström K. Wettesten M. Borén J. Bondjers G. Wiklund O. Olofsson S. O. (1986). Pulse-chase Studies of the Synthesis and Intracellular Transport of Apolipoprotein B-100 in Hep G2 Cells. J. Biol. Chem. 261, 13800–13806. 10.1016/s0021-9258(18)67090-5 3020051

[B5] Bui P. T. Hoang T. X. (2020). Protein Escape at the Ribosomal Exit Tunnel: Effect of the Tunnel Shape. J. Chem. Phys. 153, 045105. 10.1063/5.0008292 32752708

[B6] Cabrita L. D. Dobson C. M. Christodoulou J. (2010). Protein Folding on the Ribosome. Curr. Opin. Struct. Biol. 20, 33–45. 10.1016/j.sbi.2010.01.005 20149635

[B7] Chwastyk M. Cieplak M. Faisca P. F. N. (2021). Ribosome- and Chaperonin-Assisted Knotting of Proteins. (submitted).

[B8] Chwastyk M. Cieplak M. M. (2015). Cotranslational Folding of Deeply Knotted Proteins. J. Phys. Condens. Matter 27, 354105. 10.1088/0953-8984/27/35/354105 26292194

[B9] Chwastyk M. Jaskólski M. Cieplak M. (2014). Structure-based Analysis of Thermodynamic and Mechanical Properties of Cavity-Containing Proteins - Case Study of Plant Pathogenesis-Related Proteins of Class 10. FEBS J. 281, 416–429. 10.1111/febs.12611 24206126

[B10] Chwastyk M. Jaskólski M. Cieplak M. (2016). The Volume of Cavities in Proteins and Virus Capsids. Proteins 84, 1275–1286. 10.1002/prot.25076 27231838

[B11] Chwastyk M. Panek E. A. Malinowski J. Jaskólski M. Cieplak M. (2016). Properties of Cavities in Biological Structures – A Survey of the Protein Data Bank. Front. Mol. Biosci. 7, 591381. 10.3389/fmolb.2020.591381 PMC7677499 33240933

[B12] Dabrowski-Tumanski P. Piejko M. Niewieczerzal S. Stasiak A. Sulkowska J. I. (2018). Protein Knotting by Active Threading of Nascent Polypeptide Chain Exiting from the Ribosome Exit Channel. J. Phys. Chem. B 122, 11616–11625. 10.1021/acs.jpcb.8b07634 30198720

[B13] Dao Duc K. Batra S. S. Bhattacharya N. Cate J. H. D. Song Y. S. (2019). Differences in the Path to Exit the Ribosome across the Three Domains of Life. Nucl. Acids Res. 47, 4198–4210. 10.1093/nar/gkz106 30805621 PMC6486554

[B14] de Rosa R. Labedan B. (1998). The Evolutionary Relationships between the Two Bacteria *Escherichia coli* and Haemophilus Influenzae and Their Putative Last Common Ancestor. Mol. Biol. Evol. 15, 17–27. 10.1093/oxfordjournals.molbev.a025843 9491601

[B15] Elcock A. H. (2006). Molecular Simulations of Cotranslational Protein Folding: Fragment Stabilities, Folding Cooperativity, and Trapping in the Ribosome. Plos Comput. Biol. 2, e98. 10.1371/journal.pcbi.0020098 16789821 PMC1523309

[B16] Especial J. Nunes A. Rey A. Faísca P. F. (2019). Hydrophobic Confinement Modulates thermal Stability and Assists Knotting in the Folding of Tangled Proteins. Phys. Chem. Chem. Phys. 21, 11764–11775. 10.1039/c9cp01701a 31114834

[B17] Faísca P. F. N. (2015). Knotted Proteins: A Tangled Tale of Structural Biology. Comput. Struct. Biotechnol. J. 13, 459–468. 10.1016/j.csbj.2015.08.003 26380658 PMC4556803

[B18] Fischer N. Neumann P. Konevega A. L. Bock L. V. Ficner R. Rodnina M. V. (2015). Structure of the *E. coli* Ribosome-EF-Tu Complex at Å Resolution by C S-Corrected Cryo-EM. Nature 520, 567–570. 10.1038/nature14275 25707802

[B19] Frank J. Gonzalez R. L. Jr. (2010). Structure and Dynamics of a Processive Brownian Motor: the Translating Ribosome. Annu. Rev. Biochem. 79, 381–412. 10.1146/annurev-biochem-060408-173330 20235828 PMC2917226

[B20] Gabdulkhakov A. Nikonov S. Garber M. (2013). Revisiting theHaloarcula marismortui50S Ribosomal Subunit Model. Acta Crystallogr. D Biol. Cryst. 69, 997–1004. 10.1107/s0907444913004745 23695244

[B21] Gronenborn A. Filpula D. Essig N. Achari A. Whitlow M. Wingfield P. (1991). A Novel, Highly Stable Fold of the Immunoglobulin Binding Domain of Streptococcal Protein G. Science 253, 657–661. 10.1126/science.1871600 1871600

[B22] Ho B. K. Gruswitz F. (2008). HOLLOW: Generating Accurate Representations of Channel and interior Surfaces in Molecular Structures. BMC Struct. Biol. 8, 49. 10.1186/1472-6807-8-49 19014592 PMC2603037

[B23] Hoang T. X. Cieplak M. (2000). Molecular Dynamics of Folding of Secondary Structures in Go-type Models of Proteins. J. Chem. Phys. 112, 6851–6862. 10.1063/1.481261

[B24] Ingolia N. T. Lareau L. F. Weissman J. S. (2011). Ribosome Profiling of Mouse Embryonic Stem Cells Reveals the Complexity and Dynamics of Mammalian Proteomes. Cell 147, 789–802. 10.1016/j.cell.2011.10.002 22056041 PMC3225288

[B25] Kaiser C. M. Goldman D. H. Chodera J. D. Tinoco I. Jr. Bustamante C. (2011). The Ribosome Modulates Nascent Protein Folding. Science 334, 1723–1727. 10.1126/science.1209740 22194581 PMC4172366

[B26] Krobath H. Shakhnovich E. I. Faísca P. F. N. (2013). Structural and Energetic Determinants of Co-translational Folding. J. Chem. Phys. 138, 215101. 10.1063/1.4808044 23758397

[B27] Kudva R. Tian P. Pardo-Avila F. Carroni M. Best R. B. Bernstein H. D. (2018). The Shape of the Bacterial Ribosome Exit Tunnel Affects Cotranslational Protein Folding. eLife 7, e36326. 10.7554/eLife.36326 30475203 PMC6298777

[B28] Li Z. Guo Q. Zheng L. Ji Y. Xie Y.-T. Lai D.-H. (2017). Cryo-EM Structures of the 80S Ribosomes from Human Parasites *Trichomonas Vaginalis* and *Toxoplasma Gondii* . Cell Res 27, 1275–1288. 10.1038/cr.2017.104 28809395 PMC5630675

[B29] Lim K. Zhang H. Tempczyk A. Krajewski W. Bonander N. Toedt J. (2003). Structure of the YibK Methyltransferase fromHaemophilus Influenzae (HI0766): A Cofactor Bound at a Site Formed by a Knot. Proteins 51, 56–67. 10.1002/prot.10323 12596263

[B30] Liutkute M. Samatova E. Rodnina M. V. (2020). Cotranslational Folding of Proteins on the Ribosome. Biomolecules 10, 97. 10.3390/biom10010097 PMC7023365 31936054

[B31] Mallam A. L. Jackson S. E. (2011). Knot Formation in Newly Translated Proteins Is Spontaneous and Accelerated by Chaperonins. Nat. Chem. Biol. 8, 147–153. 10.1038/nchembio.742 22179065

[B32] Melnikov S. Ben-Shem A. Garreau de Loubresse N. Jenner L. Yusupova G. Yusupov M. (2012). One Core, Two Shells: Bacterial and Eukaryotic Ribosomes. Nat. Struct. Mol. Biol. 19, 560–567. 10.1038/nsmb.2313 22664983

[B33] Mioduszewski Ł. Cieplak M. (2018). Disordered Peptide Chains in an α-C-based Coarse-Grained Model. Phys. Chem. Chem. Phys. 20, 19057–19070. 10.1039/c8cp03309a 29972174

[B34] Natchiar S. K. Myasnikov A. G. Kratzat H. Hazemann I. Klaholz B. P. (2017). Visualization of Chemical Modifications in the Human 80S Ribosome Structure. Nature 551, 472–477. 10.1038/nature24482 29143818

[B35] Neidigh J. W. Fesinmeyer R. M. Andersen N. H. (2002). Designing a 20-residue Protein. Nat. Struct. Biol. 9, 425–430. 10.1038/nsb798 11979279

[B36] Nilsson O. B. Hedman R. Marino J. Wickles S. Bischoff L. Johansson M. (2015). Cotranslational Protein Folding inside the Ribosome Exit Tunnel. Cel Rep. 12, 1533–1540. 10.1016/j.celrep.2015.07.065 PMC4571824 26321634

[B37] Nissley D. A. Vu Q. V. Trovato F. Ahmed N. Jiang Y. Li M. S. (2020). Electrostatic Interactions Govern Extreme Nascent Protein Ejection Times from Ribosomes and Can Delay Ribosome Recycling. J. Am. Chem. Soc. 142, 6103–6110. 10.1021/jacs.9b12264 32138505 PMC7312765

[B38] O'Brien E. P. Christodoulou J. Vendruscolo M. Dobson C. M. (2011). New Scenarios of Protein Folding Can Occur on the Ribosome. J. Am. Chem. Soc. 133 (3), 513–526. 10.1021/ja107863z 21204555

[B39] Prosdocimi F. Zamudio G. S. Palacios-Pérez M. Torres de Farias S. V. José M. (2020). The Ancient History of Peptidyl Transferase center Formation as Told by Conservation and Information Analyses. Life 10, 134–151. 10.3390/life10080134 PMC7459865 32764248

[B40] Sehnal D. Svobodová Vařeková R. Berka K. Pravda L. Navrátilová V. Banáš P. (2013). MOLE 2.0: Advanced Approach for Analysis of Biomacromolecular Channels. J. Cheminform. 5, 39. 10.1186/1758-2946-5-39 23953065 PMC3765717

[B41] Sikora M. Sułkowska J. I. Cieplak M. (2009). Mechanical Strength of 17 134 Model Proteins and Cysteine Slipknots. Plos Comput. Biol. 5, e1000547. 10.1371/journal.pcbi.1000547 19876372 PMC2759523

[B42] Soler M. A. Rey A. Faísca P. F. N. (2016). Steric Confinement and Enhanced Local Flexibility Assist Knotting in Simple Models of Protein Folding. Phys. Chem. Chem. Phys. 18, 26391–26403. 10.1039/c6cp05086g 27722468

[B43] Sulkowska J. I. Cieplak M. (2008). Selection of Optimal Variants of Go-like Models of Proteins through Studies of Stretching. Biophys. J. 95, 3174–3191. 18567634 10.1529/biophysj.107.127233 PMC2547460

[B44] Takagi F. Koga N. Takada S. (2003). How Protein Thermodynamics and Folding Mechanisms Are Altered by the Chaperonin Cage: Molecular Simulations. Proc. Natl. Acad. Sci. 100, 11367–11372. 10.1073/pnas.1831920100 12947041 PMC208763

[B45] Trylska J. (2009). Simulating Activity of the Bacterial Ribosome. Quart. Rev. Biophys. 42, 301–316. 10.1017/s0033583510000028 20450532

[B46] Voss N. R. Gerstein M. Steitz T. A. Moore P. B. (2006). The Geometry of the Ribosomal Polypeptide Exit Tunnel. J. Mol. Biol. 360, 893–906. 10.1016/j.jmb.2006.05.023 16784753

[B47] Wołek K. Gómez-Sicilia À. Cieplak M. (2015). Determination of Contact Maps in Proteins: a Combination of Structural and Chemical Approaches. J. Chem. Phys. 143, 243105. 10.1063/1.4929599 26723590

[B48] Young R. Bremer H. (1976). Polypeptide-chain-elongation Rate in *Escherichia coli* B/r as a Function of Growth Rate. Biochem. J. 160, 185–194. 10.1042/bj1600185 795428 PMC1164221

[B49] Zhao Y. Dabrowski-Tumanski P. Niewieczerzal S. Sulkowska J. I. (2017). The Exclusive Effects of Chaperonin on the Behavior of Proteins with 52 Knotknot. Plos Comput. Biol. 14, e1005970. 10.1371/journal.pcbi.1005970 PMC5874080 29547629

